# Supplementary material for: Improved Method for Dental Pulp Stem Cell Preservation and Its Underlying Cell Biological Mechanism
Source: Cells. 2023 Aug 24;12(17):2138. doi: 10.3390/cells12172138 (PMC10486868; doi:10.3390/cells12172138)
Supplement: Supplementary file 1 [file cells-12-02138-s001.zip › cells-2491615-supplementary.pdf]

Supplementary Information

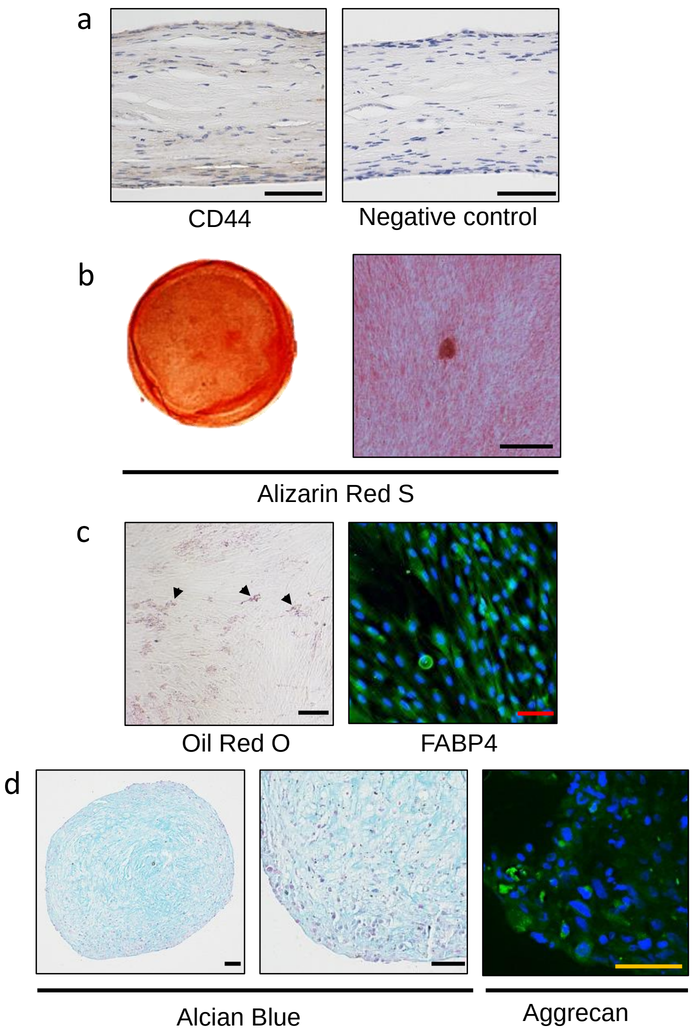

Figure S1. Characteristics of DPSCs collected in Improved CMDPT.

(a) Immunohistochemical localization of CD44 in dental pulp tissue cultured using the Improved CMDPT at 72 h. Scale bars are 50 μm. (b) Osteogenic differentiation assay. Representative images and phase-contrast micrographs after Alizarin Red S staining. Scale bar is 300 μm. (c) Adipogenic differentiation assay. Phase-contrast micrographs for Oil Red O staining (scale bar is 100 μm) and immunocytochemical localization for FABP4, a adipocyte-specific marker (scale bar is 50 μm), Arrowheads indicate the localization of Oil Red O-positive droplets. (d) Chondrogenic differentiation assay.

Representative images of Alcian Blue staining and immunohistochemical localization for aggrecan, a chondrocyte-specific marker (scale bars are 50 μm).

To confirm the characteristics of DPSCs collected in Improved CMDPT, we first immunohistochemically examined the expression of CD44, a stem cell marker, in Improved CMDPT samples after 72 hours. In addition, for the purpose of clarifying the differentiation potential of DPSCs collected in Improved CMDPT, we examined osteogenic, adipogenic and chondrogenic differentiation as in previous studies [28].

As a result, the cells localized near the upper and lower membranes in Improved CMDPT was expressed CD44, stem cell marker. This result demonstrated that DPSCs collected in Improved CMDPT had the immunophenotype of MSCs (a). In addition, after osteogenic induction for 3 weeks, DPSCs collected in Improved CMDPT formed mineralization nodules, which could be stained by Alizarin Red S (b). Moreover, after adipogenic induction for 3 weeks, DPSCs collected in Improved CMDPT formed Oil Red O-positive lipid droplets and immunocytochemically expressed FABP4 as a adipogenic marker (c). Furthermore, after chondrogenic induction for 3 weeks, DPSCs collected Improved CMDPT formed pellets. The histological findings demonstrated that the cell pellets had an Alcian Blue-positive chondrogenic matrix, and immunohistochemically, they expressed aggrecan as a chondrogenic (d). These results demonstrate that DPSCs collected in Improved CMDPT had the capacity for osteogenic, adipogenic and chondrogenic differentiation.

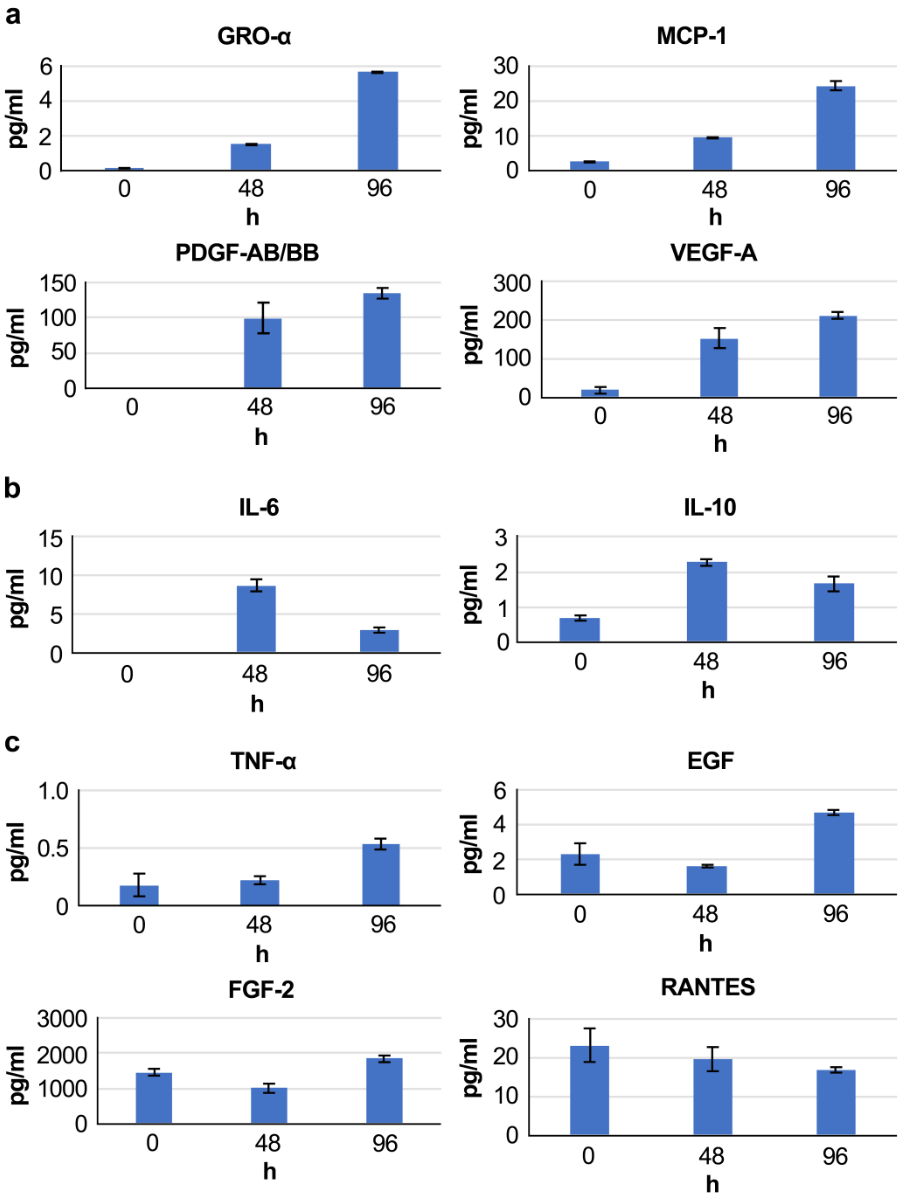

Figure S2. Identification of factors involved in cell dynamics in the Improved CMDPT. A comprehensive multiplex analysis was performed to identify molecules that may be involved in the cell dynamics of dental pulp tissue. Dental pulp tissue was homogenized at 0, 48, and 96 hours after culture using the Improved CMDPT and the supernatant was used as a sample. The target factors included the epidermal growth factor (EGF), fibroblast growth factor 2 (FGF-2), granulocyte macrophage-colony stimulating factor (GM-CSF), growth related oncogene-alpha (GRO-α), interleukin 6 (IL-6), interleukin10

(IL-10), monocyte chemoattractant protein-1 (MCP-1), platelet-derived growth factor-AA/AB (PDGF-AB/BB), regulated upon activation normal T cells expressed and presumably secreted (RANTES), tumor necrosis factor alpha (TNF-α), and vascular endothelial growth factor-A (VEGF-A). The samples were examined by multiplex using Luminex® 200 (Merck Millipore, Germany) according to the manufacturer's instructions.

(a) GRO-α, MCP-1, PDGF-AB/BB, and VEGF-A were not expressed or expressed minimally at 0 h, but increased over time. (b) IL-6 and IL-10 exhibited an increasing trend from 0 to 48 h, but their expression decreased at 96 h. (c) EGF, FGF-2, RANTES, and TNF-α did not show any change in expression over time.

In the present study, we demonstrated that SDF1 promotes DPSC migration in the Improved CMDPT. However, there are other factors that may influence migration in the Improved CMDPT based on the results of SDF1 neutralizing antibody experiments. Among the factors mentioned above, we initially focused on GRO-α and MCP-1, because these two factors are known chemotaxis factors for neutrophils and monocytes, respectively. We considered that they may also be important as cell migration-promoting factors. We plan to further examine these two factors in detail and identify additional factors in the near future.
